# Phospho*Rice*: a meta-predictor of rice-specific phosphorylation sites

**DOI:** 10.1186/1746-4811-8-5

**Published:** 2012-02-03

**Authors:** Shufu Que, Kuan Li, Min Chen, Yongfei Wang, Qiaobin Yang, Wenfeng Zhang, Baoqian Zhang, Bangshu Xiong, Huaqin He

**Affiliations:** 1 Key Laboratory of Ministry of Education for Genetic, Breeding and Multiple Utilization of Crops, Fuzhou 350002, China; 2 College of Life Sciences, Fujian Agriculture and Forestry University, Fuzhou 350002, China; 3 Key Laboratory of Nondestructive Test of Ministry of Education, Nanchang Hangkong University, Nanchang 330063, China

## Abstract

**Background:**

As a result of the growing body of protein phosphorylation sites data, the number of phosphoprotein databases is constantly increasing, and dozens of tools are available for predicting protein phosphorylation sites to achieve fast automatic results. However, none of the existing tools has been developed to predict protein phosphorylation sites in rice.

**Results:**

In this paper, the phosphorylation site predictors, NetPhos 2.0, NetPhosK, Kinasephos, Scansite, Disphos and Predphosphos, were integrated to construct meta-predictors of rice-specific phosphorylation sites using several methods, including unweighted voting, unreduced weighted voting, reduced unweighted voting and weighted voting strategies. Phospho*Rice*, the meta-predictor produced by using weighted voting strategy with parameters selected by restricted grid search and conditional random search, performed the best at predicting phosphorylation sites in rice. Its Matthew's Correlation Coefficient (MCC) and Accuracy (ACC) reached to 0.474 and 73.8%, respectively. Compared to the best individual element predictor (Disphos_default), Phospho*Rice *archieved a significant increase in MCC of 0.071 (P < 0.01), and an increase in ACC of 4.6%.

**Conclusions:**

Phospho*Rice *is a powerful tool for predicting unidentified phosphorylation sites in rice. Compared to the existing methods, we found that our tool showed greater robustness in ACC and MCC. Phospho*Rice *is available to the public at http://bioinformatics.fafu.edu.cn/PhosphoRice.

## Background

Protein phosphorylation is the most common form of protein post-translational modification (PTM) [1-3]. Phosphorylation and dephosphorylation of proteins is a universal mechanism for regulating protein function in the eukaryote, prokaryote and archaea kingdoms. Given the importance of protein phosphorylation in regulating cellular signaling, large-scale identification of phosphorylated proteins has been carried out in yeast [4], mice [5], humans [6], Arabidopsis [7,8], rice [9-12] and Medicago [13]. As the data grow, the number and the size of the available phosphoprotein databases are increasing and are becoming more complex. The Phospho.ELM database contains validated phosphorylation sites that are mostly derived from mammals [14], Phosida contains large-scale data from *Homo sapien *and *Bacillus subtilis *[15], PhosphoSite (http://www.phosphosite.org/) is a curated site that focuses on vertebrate systems [16] and PhosPhAt is a phosphorylation site database that is specific for *Arabidopsis *[17].

The growing data of protein phosphorylation sites have stimulated the development of computational approaches to predict these sites from protein sequences. Over the past decade, a series of algorithms have been developed to predict phosphorylation sites from amino acid sequences [18]. A few well-maintained web sites that offer prediction of protein phosphorylation sites have been made freely available to the scientific community, including NetPhos [19], NetPhosK [20], KinasePhos [21], KinasePhos 2.0 [22], DISPHOS [23], Scansite [24], PPSP [25], GPS [26], PredPhospho [27], NetPhosYeast [28], GANNPhos [29] and Musites [30]. However, the existing protein phosphorylation site prediction tools show a data sampling bias. The predictors perform at a high accuracy only for individual species [17]. Many existing prediction programs were primarily derived from mammalian data and exhibit poor performance in predicting plant phosphorylation sites. Therefore, based on the experimentally validated phosphorylation sites in a specific model organism, organism-specific predictors have been developed. NetPhosYeast, a yeast-specific predictor, outperforms existing generic predictors in the identification of phosphorylation sites in yeast [28]. PhosPhAt, which predicts phosphorylated-Serine sites in *Arabidopsis*, is benchmarked to perform better with Arabidopsis sequences than other generic predictors [17]. To our knowledge, no existing methods have been developed to specifically predict protein phosphorylation sites in rice.

As Arabidopsis *thaliana *(L.) standing as a model of dicotyledoneous species, rice (*Oryza sativa *L.) is a representative model monocotyledoneous (monocot) species. Moreover, rice shows an immense socio-economic impact on human civilization. In the past decade, with proteomic technologies and the availability of the genome sequences, rice proteomic research has been propelled towards a new height, which is crucial to better understand monocot plants [31]. Therefore, rice (*Oryza Sativa *L.) also serves as a cornerstone for the study of functional genomics in cereal plants [31]. However, current predictors perform poorly when individually used to predict phosphorylation sites in rice phosphoproteins [18]. In our previous research work, we constructed three different phosphorylation sites datasets to test the performance of different predictors. We found that the phosphorylation site predictors were complementary to some extent [18]. Therefore, establishment of a meta-server by maximizing complementary of individual predictors might be a promising approach to develop an improved prediction system. In this study, we developped a rice-specific meta-predictor of protein phosphorylation sites by integrating the newly individual predictors.

## Results

### Preprocessing performance assessment of element predictors

All of the protein sequences in the dataset were run through all 15 element predictors. Perl scripts were developed to submit jobs to the servers with the specified prediction options and then to analyze the prediction performance. As shown in Table 1, the element predictors showed different performances in predicting rice phosphorylation sites. The element predictor that provided the best prediction performance was Disphos_default (ACC: 69.2%, MCC: 0.403).

**Table 1:** Prediction performance of the element predictors on the test dataset

Element predictor	Sn (%)	Sp (%)	ACC (%)	MCC
KinasePhos2.0_80	81.6	51.2	65.5	0.341

KinasePhos_default	80.2	57.4	68.1	0.383
KinasePhos_90	77.0	62.3	69.2	0.395
KinasePhos_95	65.8	73.7	70.0	0.396
KinasePhos_100	37.6	89.6	65.1	0.321
Scansite_low	75.9	54.8	64.7	0.313
Scansite_middle	38.1	86.6	63.8	0.285
Scansite_high	12.8	96.5	57.1	0.173
Prephospho	95.5	13.7	52.2	0.158
DISPHOS_default	80.6	59.1	69.2	0.403
DISPHOS_ Arabidopsis	43.9	86.6	66.5	0.341
DISPHOS_ Eukaryotes	41.7	87.5	66.0	0.331
NetPhosK_0.5	75.9	46.6	60.4	0.235
NetPhosK_0.7	17.0	87.9	54.5	0.070
NetPhos2.0	70.7	59.9	65.0	0.307

Predicting performance assessed on the dataset of rice phosphorylation sites.

### Unweighted voting, unreduced weighted voting and reduced weighted voting strategies

We combined the element predictors to construct meta-predictors using unweighted voting, unreduced weighted voting and reduced weighted voting strategies. In the two-class phosphorylation site prediction problems, a score threshold must be set. The threshold score was set as half of the sum of all of the weights of the element predictors to construct meta-predictor of unweighted voting, unreduced weighted voting and reduced weighted voting strategies [32]. In this paper, the threshold scores (T) were less than half of the total weight of the predictors.

As shown in Table 2, compared to that of the best element predictors (ACC: 69.2%, MCC: 0.403), the meta-predictors constructed by unweighted voting, unreduced weighted voting and reduced weighted voting strategies achieved an significant increase in MCC of between 0.046 and 0.051. They all had a slight increase in ACC of between 3.2% and 3.7%. The meta-predictor of reduced weighted voting (with weights set by MCC) showed the best prediction performance (MCC: 0.455) in all the meta-predictors.

**Table 2:** The prediction performance of meta-predictors constructed by unweighted voting, unreduced weighted voting and reduced weighted voting strategies

predictor	ACC (%)	MCC
Best element predictor	69.2	0.403
(Disphos_default)		
Unweighted voting	72.4	0.449 (1.58E-03)*
Best unreduced weighted voting	72.5	0.450 (1.18E-03) *
(with weights set by ACC)		
Best unreduced weighted voting	72.8	0.453 (5.4E-04) *
(with weights set by MCC)		
Best reduced weighted voting	72.8	0.453 (6.0E-04) *
(with weights set by ACC)		
Best reduced weighted voting	72.9	0.454 (3.4E-04) *
(with weights set by MCC)		

* P-values in Fisher's Z-transformation test (compared with the MCC of the best element predictor) are shown in parentheses.

### Restricted grid search and Conditional random search

We also ran a weighted voting strategy with parameters selected by restricted grid search to construct meta-predictors for phosphorylation sites in rice. As shown in Table 3, we found that the weighted voting strategy with the parameters selected by restricted grid search produced a satisfactory meta-predictor, which exhibited outstanding prediction performance (ACC: 73.5%, MCC: 0.469). Compared to the best element predictor, they improved MCC of 0.066 and ACC of 4.3%.

**Table 3:** The parameters in the weighted voting meta-predictors selected by a restricted grid search and a conditional random search

Element Predictor	Parameter selected by Restricted Grid search	Random number*	Parameter selected by conditional random search
Predphospho	0	Random (1)	0
NetPhos2.0	1	Random (3)	1.23
NetPhosK_0.5	0	Random (1)	0
NetPhosK_0.7	0	Random (1)	0
KinasePhos_default	3	1+Random (4)	2.75
KinasePhos_90	1	Random (3)	2.76
KinasePhos_95	0	Random (1)	0.79
KinasePhos_100	0	Random (1)	0
DISPHOS_default	3	1+Random (4)	4.25
DISPHOS_ Eukaryotes	1	Random (3)	1.65
DISPHOS_Arabidopsis	1	Random (3)	2.22
KinasePhos2.0_80	0	Random (1)	0.71
Scansite_middle	1	Random (3)	1.6
Scansite_low	3	1+Random (4)	3.9
Scansite_high	1	Random (3)	2.57
T value	8		13.3
ACC (%)	73.5		73.8
MCC	0.469 (2.60E-06)**		0.474 (6.00E-07) **

* Random (3) means the weight could fluctuate from 0 to 3. For instance, by restricted grid search, the weight value of NetphoK 2.0 was 1, and the last grid value and next grid value were 0 and 3, respectively. In a conditional random search, the weight of Netphos 2.0 was set as random (3). The weight value of KinasePhos_default was 3, and the last grid value and next grid value were 1 and 5, respectively. Therefore, its weight was set as '1+random (4)' in a conditional random search.

** P-values in Fisher's Z-transformation test (compared with the MCC of the best element predictor) are shown in parentheses.

Following the restricted grid search, we developed a conditional random search scheme to select the value of the 16 parameters. We decided that the weight of any element predictor would be allowed to fluctuate within a certain range, which was between the last grid and the next grid of parameter selected by the restricted grid search (Table 3). For instance, the weight value of NetPhos2.0 was 1 for the restricted grid search, which last grid value was 0 and next grid value was 3. Then, in conditional random search, the weight value of NetPhosK_0.5 was set to fluctuate between 0 and 3 (Table 3). Using this strategy, we produced a conditional random search meta-predictor, which possessed the best performance than that of all the individual predictors and the meta-predictors described above (Table 3). Its MCC were 0.071 significantly higher than that of the best individual element predictor (Disphos_default), while ACC was 4.6% higher than that of the best element predictor. We named this optimal conditional random search meta-predictor Phospho*Rice*.

**Figure 1:**
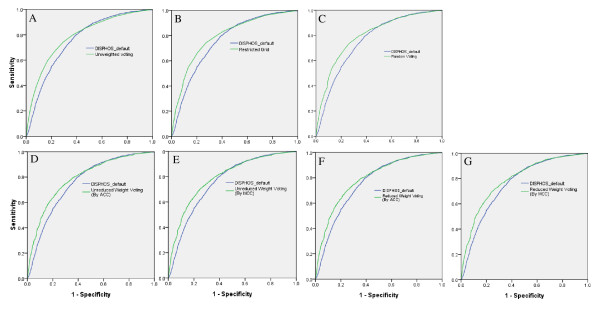
**Receiver operating characteristics curves of the prediction performance of meta predictors in comparison to that of the best element predictor (Disphos_default)**. In the diagrams, improved classification performance is indicated for predictors with increased area under the ROC. The areas under the ROC curve were showed in Table 4. A: ROC curve of unweight-voting predictor in comparison to Disphos_default. B: ROC curve of restricted-grid predictor in comparison to Disphos_default. C: ROC curve of random-voting predictor in comparison to Disphos_default. D: ROC curve of unreduced-weight-voting predictor in comparison to Disphos_default (by ACC). E: ROC curve of unreduced- weight-voting predictor in comparison to Disphos_default (by MCC). F: ROC curve of reduced- weight-voting predictor in comparison to Disphos_default (by ACC). G: ROC curve of reduced- weight-voting predictor in comparison to Disphos_default (by MCC). * By ACC: the weights of meta-predictor were selected to result in the optimal ACC; By MCC: the weights of meta-predictor were selected to result in the optimal MCC.

Moreover, we generated the receiver operating characteristic (ROC) curve according to the predicted potentials of meta predictors. ROC is a plot of the true-positive ratio (sensitivity) against the false-positive ratio (1-specificity). The area under an ROC curve (AUC) represents the trade-off between sensitivity and specificity. The ROC curves of the prediction performance of all the meta-predictors in comparison to that of the best element predictor (Disphos_default) were shown in Figure 1. All meta-predictors had higher ROC areas than that of the best element predictor (Table 4). Meanwhile, we calculated the area underneath ROC curve to compare the predicting performance of PhosphoRice with that of Musite. Musite was a Java-based standalone application for predicting both general and kinase-specific protein phosphorylation sites [30]. Table 5 showed that the performance of PhosphoRice was significantly higher than that of Musite (Table 5).

**Table 4:** Areas under the ROC curves for the best element predictor, meta-predictors constructed by unweighted voting, unreduced weighted voting, reduced weighted voting and weighted voting strategies.

Predictor	Area
Best element predictor	0.758
(Disphos_default)	
Unweighted voting	0.788
Best unreduced weighted voting	0.791
(with weights set by ACC)	
Best unreduced weighted voting	0.792
(with weights set by MCC)	
Best reduced weighted voting	0.791
(with weights set by ACC)	
Best reduced weighted voting	0.791
(with weights set by MCC)	
Weighted voting	0.794
(By restricted grid search)	
A combination of weight voting and random	0.796

**Table 5:** The prediction performance of Phospho*Rice *in comparison to that of Musite

*Predictor*	*ACC (%)*	*MCC*	*Area*
PhosphoRice	72.4	0.474 (0.044) *	0.796
Musite	73.8	0.446	0.793

* P-value in Fisher's Z-transformation test (compared with the MCC of Musite) is shown in parenthes.

## Discussion

### Prediction performance of element predictors

Before being integrated into the meta-predictors, the existing phosphorylation site predictors used in this study were tested and assessed on the rice phosphorylation site dataset. All of element predictors achieved an ACC over 50.0%. However, their MCC was quite difference from each other, which was between 0.07 and 0.403. Different predictors may yield different performance in phosphorylation sites prediction due to their different types of algorithm and training dataset. The result also showed that some of kinase family-specific predictors could yield good performance under no kinase-specific condition, such as KinasePhos_95 (ACC: 70.0%, MCC: 0.396).

### Prediction performance of unweighted voting, unreduced weighted voting and reduced weighted voting meta-predictors

In this paper, the prediction performance of unweighted voting, unreduced weighted voting and reduced weighted voting meta-predictors exceeded that of the best element predictor (ACC: 69.2%, MCC: 0.403), showing a significant increase in MCC (P < 0.01). The good performance archieved by these meta-predictors was due to element predictors' complementing each other. The reduced weighted voting strategies had been applied to produce meta-predictors in protein subcellular localization prediction [33] and phosphorylation site prediction for specific kinase family [32]. However, it got different result. This strategy produced good meta-predictors in the protein subcellular localization prediction problem [33], but failed to yield meta-predictors with expected performance in the prediction of phosphorylation sites for the CK2 kinase family [32]. Wan et al. (2008) discussed that the stronger correlation among the element predictors might play a role for the failure. However, we argued that the selection of element predictors was vital to the prediction performance of meta-predictors. The prediction performance of six element predictors used in this study was evaluated in Que et al. (2010). We found that the element predictors were complementary to some extent.

### Prediction performance of Phospho*Rice*

In this study, we applied a more general form of the weighted voting strategy. First, we used a restricted grid search to determine a range for the parameters. Second, we set ranges of the parameters selected by the restricted grid search to perform a conditional random search. The restricted grid search was very efficient in running time performance and in parameter selection. It has been widely used to construct meta-predictors, including a serine/threonine phosphorylation site predictor [32] and a protein-protein interaction site predictor [34]. Using the restricted grid search, we selected 9 non-zero weight parameters for the final meta-predictors (Table 3). However, a drawback of using a restricted grid search is that it might find a local, rather than a global, optimum. Therefore, based on the result of restricted grid search, we ran an exhaustive search approach, conditional random search, to determine the 16 parameters. The conditional random search produced a good meta-predictor, whose rice phosphorylation site prediction performance not only exceeded that of the best element predictor, but also surpassed that of the meta-predictors integrated with unweighted voting, unreduced weighted voting and reduced weighted voting strategies. We can conclude here that a combined restricted grid search and conditional random search may be a good approach for determining the parameters in weighted voting strategy.

## Conclusion

To summarize, we created a meta-predictor, Phospho*Rice*, using a weighted voting strategy, in which parameters were selected by restricted grid search and conditional random search. It shows good performance in predicting rice phosphorylation sites, as measured by the MCC and ACC. Its MCC were 0.071 significantly higher than that of the best individual element predictor (Disphos_default), while ACC was 4.6% higher than that of the best element predictor. We have also provided a web service for the prediction of rice protein phosphorylation sites, which can be accessed at http://bioinformatics.fafu.edu.cn/PhosphoRice.

## Methods

### Preprocessing of dataset

We collected rice phosphorylation sites from recent literature, including Nakagami *et al*. (2010), and the feature table of Swiss-Prot database. After removing the redundant phosphorylation sites, the number of serine (S), threonine (T) and tyrosine (Y) substrates were 4220, 605 and 141 respectively (Table 6). These phosphorylation sites were involved in 2162 proteins (Additional file 1). The 25-mer sequences (-12 ~ +12) of phosphorylation sites were extracted from the protein sequences and constructed as dataset. Because all of the phosphorylation sites in the positive dataset were experimentally verified, they were regarded as (+) sites. The Ser, Thr and Tyr residues that were not annotated as phosphorylation sites within the dataset were regarded as (-) sites (*i.e*., non-phosphorylation sites). We balanced the positive and negative dataset and the sizes of positive dataset and negative dataset are equal during cross-validation processes (Table 6).

**Table 6:** Number of phosphoserine, phosphothreonine and phosphotyrosine sites in positive and negative dataset

*Dataset *	*Number of phosphorylation sites*			Total
		
	Serine	Threonine	Tyrosine	
Positive dataset	4220	605	141	4966

Negative dataset	2954	1798	834	5586

We used a standard 10-fold cross validation to optimize the weight of all the individual predictors, and calculated the ACC and MCC of each meta predictor. The dataset was randomly partitioned into 10 subsets, including one testing subset and nine training subsets. The weights are updated and the ACC and MCC were recalculated. The new weights were kept only if the ACC and MCC increased; otherwise the weights are rolled back to the previous values. Using this strategy, the meta-predictors were training by shifting the test subset stepwise so that all data is used for training and test when completed.

### Selection of element predictors

Six phosphorylation site prediction programs, NetPhosK, NetPhos2.0, KinasePhos, PrePhospho 1.0, Scansite and DISPHOS, were selected as elemental predicting programs. NetPhosK, KinasePhos, PrePhospho 1.0 and Scansite are kinase-family-specific phosphoryaltion site predictor, while NetPhos2.0 and DISPHOS are not. All of the element predictors were run under no kinase-specific condition. Their prediction performance was evaluated in our last research work. Fifteen element predictors derived from these programs were used to form rice-specific meta-predictors of phosphorylation sites (Additional file 2). The methods for obtaining these 15 element predictors are described below.

Netphos and NetPhosK (http://www.cbs.dtu.dk/services/NetPhosK/) use an artificial neural network algorithm to predict phosphorylation sites. With the NetPhosK prediction server, the option "prediction without filtering" was selected to predict phosphorylation sites. The threshold value was set as 0.5 and 0.7 to determine whether or not a site is predicted as phosphorylated. The result at each threshold value was selected to be an element predictor, they were named NetPhosK_0.5 and NetPhosK_0.7.

DISPHOS (DISorder-enhanced PHOSphorylation site predictor, http://core.ist.temple.edu/pred/) uses position-specific amino acid composition and predicts structural disorder information to distinguish phosphorylation and non-phosphorylation sites. In this study, "default predictor," "Eukaryotes" or "*A. thaliana*" was chosen to predict phosphorylation sites in rice and were named Disphos_default, Disphos_Eukaryotes and Disphos_Arabidopsis, respectively.

KinasePhos (http://kinasephos.mbc.nctu.edu.tw/index.php) employs a Profile Hidden Markov Model (HMM) to predict kinase family-specific phosphorylation sites. In this study, KinasePhos was run with the option of 90%, 95%, 100% prediction specificity and 'by default HMM bit score', whilst KinasePhos 2.0 with 80% prediction specificity, respectively. These five selections resulted in four separate element predictors termed KinasePhos_90, KinasePhos_95, KinasePhos_100, KinasePhos_default and KinasePhos 2.0_80.

Scansite (http://scansite.mit.edu/) uses scores calculated from position-specific score matrices (PSSM) to search for motifs within proteins that are likely to be phosphorylated by specific protein kinases. In this work, the setting of a high, medium or low stringency level was selected and resulted in the production of three separate element predictors named Scansite_high, Scansite_medium and Scansite_low, respectively.

PredPhospho (http://pred.ngri.re.kr/PredPhospho.htm) predicts various kinase-specific phosphorylation sites by training SVMs. In this study, the prediction was made by considering all kinase groups and families.

### Prediction and performance measures

It was difficult to compare the numerical scores produced by the individual element predictors due to their differences in mathematical meaning [32]. In this study, the value of the scores was ignored, and instead a binary value was assigned (representing phosphorylated or not phosphorylated) and then performance was compared across prediction programs.

Four measurements-Sensitivity (*Sn*), Specificity (*Sp*), Accuracy (*ACC*) and the Matthew's Correlation Coefficient (*MCC*)-were employed to evaluate the performance of the tested predictors (definitions below):

$$\begin{array}{c} Sn=\frac{TP}{TP+FN},\\ Sp=\frac{TN}{TN+FP},\\ Ac=\frac{TP+TN}{TP+FP+TN+FN}, \end{array}$$

and

$$MCC=\frac{\left(TP\times TN\right)-\left(FN\times FP\right)}{\sqrt{\left(TP+FN\right)\times \left(TN+FP\right)\times \left(TP+FP\right)\times \left(TN+FN\right)}}.$$

where *TP*, *FP*, *FN*, and *TN *denote true positives, false positives, false negatives, and true negatives. *Sn *and *Sp *illustrate the correct prediction ratios of positive and negative datasets, respectively. Because MCC is much less susceptible to the ratio of positive samples and negative samples in the dataset, it is the most widely used prediction measure for two-class prediction programs [32].

We used SPSS 16.0 to create operating characteristic (ROC) curves to measure the performance of meta-predictors. For each possible threshold, the sensitivity and specificity were evaluated, the ROC curve [sensitivity versus (1-specificity) curve] was plotted, and the area underneath this curve was calculated. In this study, ROC curves were used to compare the predicting performance of every meta-predictors with the best element predictor, Disphos_default, respectively. The area underneath ROC curve was calculated to compare the predicting performance of PhophoRice with Musite, which was a newly predictor.

### Unweighted voting, unreduced weighted voting and reduced weighted voting strategies

The unweighted voting, unreduced weighted voting and reduced weighted voting strategies were used to construct meta-predictors according to the procedure outlined by Liu *et al*. (2007) and Wan *et al*.(2008). Generally, if the following condition was satisfied, a linear voting-based two-class classifier would make a positive prediction:

(1) $$\overset{N}{\underset{j=1}{\sum }}\left[P_{j}\cdot w_{j}\right]\geq T$$

Where N is the total number of element predictors (in this experiment, N = 15), w_j _is the weight of the jth prediction method and w_j _= 1 for all element predictors in the unweighted voting strategy. P_j _is the prediction made by the jth predictor; in a positive prediction, P_j _= 1, otherwise P_j _= 0. T is the threshold score.

For a simple weighting voting strategy, the threshold T can be set as the half of the total weight of the predictors.

(2) $$\text{T}=\frac{1}{2}\overset{\text{N}}{\underset{\text{j}=1}{\sum }}{\text{w}}_{\text{j}}$$

### Restricted grid search

In Equation (1), proper weight parameters (w_j_) would produce a classifier with good prediction performance. In this study, there are 16 parameters, including 15 possible values for w_j_, and a value for T that needs to be determined for the highest performance classifier. We applied the restricted grid search method to select the values of these 16 parameters, which has been widely used in two-class classification problems [32,33]. There were two critical restrictions of this method in our study. First, we limited the weight of the element predictors to be one of the following values: 0, 1, 3, 5, 7, 9, 11, 13, and 15. Second, the sum of the weights of all 15 element predictors must be equal to 15 (Table 7). The restricted grid search of the 16 parameters was conducted on the dataset with 10-fold cross-validation.

**Table 7:** Weight combinations, permutations and possible weights sum values in the restricted grid search scheme

Weight combinations*	Number of corresponding weight**
15 × (1)	$P_{15}^{1}$ = 15
1 × (2)+13 × (1)	$P_{15}^{2}\times P_{13}^{1}$ = 1365
1 × (1)+3 × (1)+11 × (1)	$P_{15}^{1}\times P_{14}^{1}\times P_{13}^{1}$ = 2730
1 × (4)+11 × (1)	$P_{15}^{4}\times P_{11}^{1}$ = 15015
1 × (1)+5 × (1)+9 × (1)	$P_{15}^{1}\times P_{14}^{1}\times P_{13}^{1}$ = 2730
3 × (2)+9 × (1)	$P_{15}^{2}\times P_{13}^{1}$ = 1365
1 × (3)+3 × (1)+9 × (1)	$P_{15}^{3}\times P_{12}^{1}\times P_{11}^{1}$ = 60060
1 × (6)+9 × (1)	$P_{15}^{6}\times P_{9}^{1}$ = 45045
1 × (1)+7 × (2)	$P_{15}^{1}\times P_{14}^{2}$ = 1365
3 × (1)+5 × (1)+7 × (1)	$P_{15}^{1}\times P_{14}^{1}\times P_{13}^{1}$ = 2730
1 × (3)+5 × (1)+7 × (1)	$P_{15}^{3}\times P_{12}^{1}\times P_{11}^{1}$ = 60060
1 × (2)+3 × (2)+7 × (1)	$P_{15}^{2}\times P_{13}^{2}\times P_{11}^{1}$ = 90090
1 × (5)+3 × (1)+7 × (1)	$P_{15}^{5}\times P_{10}^{1}\times P_{9}^{1}$ = 270270
1 × (8)+7 × (1)	$P_{15}^{8}\times P_{7}^{1}$ = 450450
5 × (3)	$P_{15}^{3}$ = 455
1 × (2)+3 × (1)+5 × (2)	$P_{15}^{2}\times P_{13}^{1}\times P_{12}^{2}$ = 90090
1 × (5)+5 × (2)	$P_{15}^{5}\times P_{10}^{2}$ = 135135
1 × (1)+3 × (3)+5 × (1)	$P_{15}^{1}\times P_{14}^{3}\times P_{11}^{1}$ = 60060
1 × 4+3 × 2+5 × 1	$P_{15}^{4}\times P_{11}^{2}\times P_{9}^{1}$ = 675675
1 × (7)+3 × (1)+5 × (1)	$P_{15}^{7}\times P_{8}^{1}\times P_{7}^{1}$ = 360360
1 × (10)+5 × (1)	$P_{15}^{10}\times P_{5}^{1}$ = 15015
3 × (5)	$P_{15}^{5}$ = 3003
1 × (3)+3 × (4)	$P_{15}^{3}\times P_{12}^{4}$ = 225225
1 × (6)+3 × (3)	$P_{15}^{6}\times P_{9}^{3}$ = 420420
1 × (9)+3 × (2)	$P_{15}^{9}\times P_{6}^{2}$ = 75075
1 × (12)+3 × (1)	$P_{15}^{12}\times P_{3}^{1}$ = 1365
1 × (15)	$P_{15}^{15}$ = 1
Possible weighted	0, 1, 2, 3, 4, 5, 6, 7, 8, 9, 10, 11, 12, 13, 14, 15

* Weight combinations are denoted as the sum of each weight value multiplied by the number of weights taking the weight value, with the weight value = 0 omitted.

** For instance, "15 × (1)"represents that 1 of the 15 weights takes the value 15, and the other 14 weights take the value 0; and "1 × (1)+3 × (1)+11 × (1)" represents that 1 of the 15 weights takes the value 1, 1 weight takes the value 3, 1 weight takes 11 and the remaining 12 weights take the value 0. Each weight combination corresponds to one or more weight permutations. For instance, for weight combination "15 × (1)," the weight value 15 can be taken by each of the 15 weights; thus, it corresponds to $P_{15}^{1}$ weight permutations.

### Conditional random search

Conditional random fields were first introduced by Lafferty and colleagues in 2001 [35]. For the conditional random search, the threshold T was set as a random value of the total weight of the predictors.

(3) $$T=rand\left(\overset{N}{\underset{j=1}{\sum }}w_{j}\right)$$

Randomized algorithms are often simple, beautiful and efficient for selecting parameters. They produce a series of unrelated and unpredictable digits or characters. However, the computer cannot produce an absolute random number; it can only have a "pseudorandom number". The conditional random search method can be represented as follows:

a. the weight selected by restricted grid search;

b. random search range was set between the last grid and the next grid of parameter selected by the restricted grid search;

c. runuing random search program;

d. training on the training set, test on the test set;

e. stopping at the parameter combination that achieve higher MCC than that of restricted grid search.

## Competing interests

The authors declare that they have no competing interests.

## Authors' contributions

HQH conceived of the study, designed experiments, analyzed data and revised the manuscript. SFQ designed and carried out restricted grid and random search. KL developed Perl scripts. MC analyzed on the performance of element and meta predictors. QBY constructed the dataset. YFW participated in the dataset construction. WFZ and BQZ developed and maintained the website. BSX helped to write the computer program. All authors read and approved the final manuscript.

## Supplementary Material

Additional file 1 **Rice phosphorylation sites data**. Data file listing Accession Number, full-length sequence, phosphorylated amino acid and its site position. Click here for file

Additional file 2 **Summary of the 15 element predictors**. Summary file listing the name, references and URLs of the 15 element predictors used to produce meta-predictors. Click here for file
