# Supplementary material for: PhosphoRice: a meta-predictor of rice-specific phosphorylation sites
Source: Plant Methods. 2012 Feb 3;8:5. doi: 10.1186/1746-4811-8-5 (PMC3395875; doi:10.1186/1746-4811-8-5)
Supplement: Additional file 2 — Summary of the 15 element predictors. Summary file listing the name, references and URLs of the 15 element predictors used to produce meta-predictors. [file 1746-4811-8-5-S2.DOC]

| Element predictor | References | URLs |
| --- | --- | --- |
| NetPhos2.0 | (Blom et al. 1999) | http://www.cbs.dtu.dk/services/NetPhos/ |
| NetPhosK_0.5 | (Blom et al. 2004) | http://www.cbs.dtu.dk/services/NetPhosK/ |
| NetPhosK_0.7 |
| KinasePhos 2.0_80 | (Wong et al. 2007) | http://kinasephos2.mbc.nctu.edu.tw/ |
| KinasePhos_90 | (Huang et al. 2005) | http://kinasephos.mbc.nctu.edu.tw/ |
| KinasePhos_95 |
| KinasePhos_100 |
| KinasePhos_default |
| Predphospho | (Kim et al. 2004) | http://www.nih.go.kr/predphospho/proteo/html /inc_PredPhospho.htm;  <http://pred.ngri.re.kr/PredPhospho.htm> |
| Scansite_low | (Obenauer et al. 2003) | http://scansite.mit.edu/motifscan_seq.phtml |
| Scansite_mid |
| Scansite_high |
| DISPHOS_default | (Iakoucheva et al. 2004) | http://www.ist.temple.edu/disphos/ |
| DISPHOS _Eukaryotes |
| DISPHOS _Arabidopsis |
